# Brief mind–body exercise in high-latitude aging: reduced sedentarism and domain-specific cognitive/social benefits

**DOI:** 10.3389/fpubh.2025.1725846

**Published:** 2026-01-13

**Authors:** Ruby Méndez Muñoz, Miguel Fuentes Contreras, Matías Castillo-Aguilar, Pablo Valdés-Badilla, Jordan Hernandez-Martinez, Oscar Adolfo Niño Méndez, Cristian Núñez-Espinosa

**Affiliations:** 1 Escuela de Medicina, Universidad de Magallanes (UMAG), Punta Arenas, Chile; 2 Centro Asistencial Docente y de Investigación (CADI-UMAG), Punta Arenas, Chile; 3 Carrera de Fonoaudiología, Universidad de Magallanes (UMAG), Punta Arenas, Chile; 4 Department of Physical Activity Sciences, Faculty of Education Sciences, Universidad Católica del Maule, Talca, Chile; 5 Sports Coach Career, Faculty of Life Sciences, Universidad Viña del Mar, Viña del Mar, Chile; 6 Department of Physical Activity Sciences, Universidad de Los Lagos, Osorno, Chile; 7 Department of Education, Faculty of Humanities, Universidad de la Serena, La Serena, Chile; 8 Centro de Investigación en Actividad Física, Ejercicio y Deporte (CAFED), Facultad de Ciencias del Deporte y la Educación Física, Universidad de Cundinamarca, Cundinamarca, Colombia

**Keywords:** aging, cognition, mind–body therapies, motor activity, polar regions, sedentary behavior

## Abstract

**Objective:**

Older adults living at high southern latitudes face compounded barriers to mobility and social participation due to cold, wind, and seasonality. Visuospatial–executive functions, which support everyday tasks such as route finding, dual-task walking, and planning in older adults, are particularly vulnerable to age-related decline after the seventh decade of life. Low-dose, climate-adapted mind–body exercise (MBE), delivered once weekly in community settings, may offer a feasible way to reduce sedentary time and support cognitive and social functioning, yet evidence from sub-Antarctic settings is scarce. We tested whether a short, standardized MBE program produces preliminary changes in sedentary behavior, physical activity, cognition, and socio-affective outcomes.

**Materials and methods:**

In a pre-experimental single-group pre–post design, community-dwelling older people (*N* = 44; Punta Arenas, Chile; mean age 69.7 ± 5.3 años) completed 24 supervised sessions (60 min, 1/week) across 24 weeks. Outcomes were IPAQ-Short (MET-min/week; sitting min/day), cognition (MoCA), and empathy (Interpersonal Reactivity Index, IRI). Bayesian linear mixed-effects models estimated pre–post changes and moderation by age, sex, and education.

**Results:**

Sitting time decreased (*β* = −0.71; 95% CrI −2.07 to 0.52; pd = 86.1%), and weekly physical activity increased (*β* = 0.52; −0.76 to 1.81; pd = 78.9%). Age moderated sedentariness change (age × time *β* = 0.46; 0.03 to 0.89; pd = 98%: relatively younger participants reduced sitting more). Global MoCA remained stable (*β* = −0.27; −1.63 to 1.10; pd = 65.2%), while the visuospatial–executive domain tended to improve (*β* = 0.45; −0.87 to 1.78; pd = 74.5%). Empathy (IRI total) increased (*β* = 0.59; −0.75 to 1.94; pd = 81.2%) with a sex interaction (sex × time *β* = −0.97; −1.92 to −0.07; pd = 97.8%: women improved more).

**Conclusion:**

A brief, indoor, regionally adapted MBE program is feasible and produces a meaningful profile of benefits, including reduced sedentarism, selective enhancement of executive-visuospatial function, and increased empathy, in older people living at high southern latitudes.

## Introduction

Preserving cognitive and functional independence in aging populations is a global health priority, particularly as challenges like cognitive decline and social disengagement intensify (1). Interventions such as multicomponent training and mind–body exercise (MBE) have emerged as promising strategies, demonstrating consistent benefits for executive functions, postural balance, and processing speed (2, 3). A key advantage of these programs is their ability to significantly reduce sedentary behavior, a primary modifiable risk factor for poor cardiometabolic and brain health (4). However, the effects of short-term interventions are often subtle, manifesting in specific cognitive subdomains before impacting global scores, and are highly sensitive to environmental and behavioral moderators (5).

However, response to the intervention can vary due to interindividual differences in baseline status, cognitive reserve, physical activity level, and contextual factors, which may dilute acute effects (6). This heterogeneity, together with the limitations of conventional cognitive assessments, complicates the interpretation of global scores; accordingly, it is more informative to focus on sensitive subdomains and to complement them with behavioral metrics, using Bayesian estimation to characterize uncertainty. Emerging evidence indicates that initial improvements are more reliably detected in visuospatial-executive functions and foundational regulatory systems, such as postural control, than in broad cognitive metrics (7). These targeted gains, coupled with the cardiometabolic benefits of increased physical activity, may lay the groundwork for more substantial cognitive enhancements if new behaviors are sustained over time (4, 8, 9).

These challenges are acutely amplified for older people in high-latitude austral regions. Severe environmental stressors, including extreme seasonality, persistent cold, and high winds, turn outdoor interactions into an unattractive activity, disrupt routines, and constrain social life (10). Such conditions can directly interfere with the neurobiological and socio-affective regulation of aging due to the lack of groups engaging in outdoor activities or individual activities under these conditions. For instance, recent findings from the sub-antarctic Magallanes region show that markers of brain plasticity, like Brain-Derived Neurotrophic Factor (BDNF), are seasonally vulnerable during winter, even in individuals with protective factors such as high cognitive reserve (11). This highlights a pressing need for interventions designed for climatic adversity and analytical methods that can capture heterogeneous outcomes (5).

This confluence of factors reveals a critical gap. Despite the compelling biological rationale for physical exercise, no sustainable, evidence-based solutions have been validated to effectively confront sedentarism and preserve cognitive health in older people living in these uniquely challenging environments. Climate-related and accessibility barriers consistently undermine program adherence, while the available research remains sparse and often relies on global measures insensitive to brief interventions (10, 12). Consequently, a significant knowledge gap persists regarding effective health strategies for this vulnerable population.

To address this gap, this study aimed to evaluate the impact of a standardized, 24-session, once-weekly MBE program on sedentarism and cognitive/social behavior in older people living in a high-latitude southern region. Given that the total weekly exercise dose is intentionally low compared with international recommendations, we framed the study as a feasibility-oriented, proof-of-concept evaluation of whether such a minimal, climate-adapted program can elicit detectable changes in self-reported sedentary time, cognitive performance, and socio-affective dimensions such as empathy. Rather than testing a full public-health solution, our goal was to generate data that could guide the design of more intensive and scalable interventions in extreme environmental contexts.

## Materials and methods

### Study design

This study utilized a pre-experimental, single-group, pre-test/post-test design. A cohort of 44 older people was recruited from the CADI-UMAG Teaching and Research Center (Centro Asistencial Docente e Investigación) to participate in the intervention. We planned a feasibility-stage sample of 40–45 people using conventional paired pre–post power to ensure interpretable within-person change. Assuming a two-sided *α* = 0.05 and a baseline–post correlation of *r* = 0.50, this sample affords ~80% power to detect a moderate standardized mean change (Cohen’s dz ≈ 0.40–0.45) in the primary behavioral outcomes, and yields an estimation precision of ~0.30 SD half-width for the 95% interval around the standardized change. Allowing for ~15% attrition, power remains ≥ ~0.75 (*n* ≈ 38), which is acceptable for feasibility aims. While planning used frequentist conventions for transparency, the primary inference is Bayesian, under weakly informative priors on the standardized change. Accordingly, we report standardized effects with 95% credible intervals and probability of direction, aligning the planning threshold with a practically meaningful posterior decision region. A cohort of 44 community-dwelling older adults was enrolled and assessed at baseline and post-intervention after a 24-week program. All participants received the same intervention (no parallel control group), and analyses focused on within-participant change over time.

### Participants

The sample consisted of 44 older people residing in a high-latitude southern city (Punta Arenas, Chile). Participants were recruited through non-probabilistic convenience sampling within the framework of a physical-cognitive stimulation program. All individuals provided written informed consent after receiving a detailed explanation of the research objectives and procedures. Eligible participants were community-dwelling adults ≥60 years, able to ambulate independently (assistive devices permitted) and follow multistep instructions, without a prior clinical diagnosis of dementia or other major neurocognitive disorder, and screen-negative for severe cognitive impairment, on stable medical treatment for ≥4 weeks, and available to attend once-weekly sessions over 24 weeks and complete pre/post-assessments. Exclusion criteria were contraindications to moderate-intensity exercise, unstable cardiovascular, metabolic, or pulmonary conditions (e.g., recent myocardial infarction or stroke <6 months, decompensated heart failure, uncontrolled arrhythmias, severe valvular disease, uncontrolled hypertension, SBP ≥ 140 mmHg or DBP ≥ 100 mmHg, or unstable angina), musculoskeletal conditions precluding safe participation, current enrollment in a similar supervised mind–body program; severe, unstable psychiatric disorder or recent substance use disorder (<6 months), sensory deficits that prevented following instruction despite correction; and planned relocation or prolonged absence during the 24-week period. Adherence was monitored via attendance logs, with the per-protocol set defined *a priori* as ≥80% attendance and the intention-to-treat set including all participants with at least one post-baseline assessment, noting that adherence thresholds were not used to exclude candidates at enrollment.

### Procedure

Each participant completed 24 supervised sessions of a mind–body exercise (MBE) program, one 60-min group session per week for 24 weeks, integrating conscious breathing and slow, coordinated movement with the progressive, safe development of muscle strength, mobility, balance, and functional control. The program was deliberately designed as a low-dose, indoor option compatible with climatic constraints, emphasizing light-to-moderate effort that allowed participants to maintain conversation and execute movements with precision and postural control, rather than targeting specific heart-rate zones. Exercises were organized in blocks (see Table 1) that combined sitting and standing tasks, dual-task elements (coordinated upper- and lower-limb patterns), and brief mindful breathing or body-scan segments. To ensure fidelity, instructors followed a standardized procedures manual and session checklists that specified the sequence, duration, and qualitative cues for each block; attendance was recorded to verify ≥80% adherence.

**Table 1:** Overview of the 24-week mind–body exercise (MBE) program: session blocks, phase-specific content, primary focus, and progression.

Session block	Weeks 1–8 (phase I/adaptation)	Weeks 9–16 (phase II/consolidation)	Weeks 17–24 (phase III/progressive)	Task type	Primary focus	Progression features
Centering and diaphragmatic breathing (5 min)	Seated posture, basic diaphragmatic breathing (4–5 s inhale/4–5 s exhale), eyes open; simple body-scan cues.	Longer breathing cycles (6–7 s), introduction of brief breath holds, option to close eyes; guided body-scan with focus on trunk and lower limbs.	Extended cycles (7–8 s), brief paced breathing with counting tasks; alternating internal (body) and external (sound/room) focus.	Low-intensity mind–body	Breathing, interoceptive attention, emotional regulation	↑ Breathing cycle length; ↑ complexity of attentional cues; gradual shift from external to internal focus.
Joint mobility and dynamic warm-up (10 min)	Seated and standing mobility for neck, shoulders, hips, knees, and ankles; slow marching in place with arm swings.	Multi-planar movements (diagonal arm patterns, hip circles) combined with slow walking; inclusion of gentle trunk rotation.	Continuous multi-directional walking patterns with coordinated arm movements (cross-body reaches), small changes in speed and direction.	Low–moderate dynamic mobility	Movement awareness, joint range, preparatory activation	↑ Range of motion; ↑ continuity of movement; mild ↑ in speed and step amplitude.
Multi-planar balance and gait patterns (20 min)	Basic tandem stance near a chair, wide-base stance, slow forward walking; simple weight shifts (front–back, side–side).	Narrow-base stance, semi-tandem and tandem walking; introduction of obstacle stepping and gentle direction changes.	Single-leg stance with support as needed; tandem walking with head turns, dual-task elements (counting, naming), larger directional changes.	Dynamic balance and gait	Balance, postural control, divided attention	↓ Base of support; ↑ time in single-leg stance; ↑ dual-task complexity; ↑ variability of direction.
Chair-based strengthening (15 min)	Sit-to-stand from standard chair, seated knee extensions, ankle plantarflexion, gentle marching; 1–2 sets of 8–10 repetitions.	Added elastic bands for knee extension/abduction; mini-squats holding chair; 2 sets of 10–12 repetitions.	Deeper sit-to-stand and mini-squats, combined arm–leg patterns with bands; 2–3 sets of 12–15 repetitions as tolerated.	Strength/functional tasks	Lower-limb and trunk strength, functional capacity	↑ Sets and repetitions; ↑ resistance (bands); ↑ complexity via arm–leg coordination.
Mindfulness-oriented cool-down and imagery (10 min)	Seated relaxation, slow breathing, brief guided imagery (e.g., pleasant landscape); focus on muscle relaxation of legs and shoulders.	Longer guided imagery scripts emphasizing body–breath synchrony; brief gratitude or positive emotion prompts.	Alternating short silent periods and guided cues; imagery integrating previous movement sequences; emphasis on transferring calm attention to daily life.	Low-intensity mindfulness	Breathing, sustained attention, emotional regulation	↑ Duration of silent practice; ↑ imagery richness; ↑ emphasis on self-directed practice.

Primary behavioral outcomes were physical activity (IPAQ-Short: MET-min/week) and sedentary behavior (sitting minutes/day), measured at baseline and post-intervention. Cognitive outcomes included global cognition and subdomains assessed with (MoCA). Secondary outcomes included socio-affective function (empathy, IRI instrument). Covariates (age, sex, years of education, and IPAQ activity level: Low/Moderate/High) were prespecified for adjusted analyses.

### Instruments

Three primary instruments were used for data collection: a sociodemographic information sheet, the International Physical Activity Questionnaire (IPAQ, short form), and the Montreal Cognitive Assessment (MoCA, Spanish version). Trained evaluators administered all instruments.

### Sociodemographic and health information

At baseline, we collected age, sex, years of education, marital status, living arrangement, and self-reported medical diagnoses and medications using a structured interview administered by trained staff.

### International Physical Activity Questionnaire (IPAQ)

The short-form Spanish version of the International Physical Activity Questionnaire (IPAQ) was used to assess physical activity levels. This self-report instrument categorizes individuals into low, moderate, or high physical activity levels based on the frequency, duration, and intensity of physical activities performed over the past 7 days. Weekly metabolic equivalents (MET-min/week) were calculated according to standardized protocols (13). Although the IPAQ-Short was originally designed for adults up to 69 years of age, it remains widely used in community-dwelling older populations, and Spanish-language validation studies in Latin-American adults support its feasibility and acceptable reliability as a pragmatic indicator of physical activity in this age range. The instrument has demonstrated acceptable reliability (test–retest ICC = 0.62) in the Chilean adult population (14), and its Spanish version has shown good internal consistency and cultural adaptation, supporting its use in Spanish-speaking populations (15).

### Montreal Cognitive Assessment (MoCA)

Cognitive function was evaluated using the Montreal Cognitive Assessment (MoCA) (16). The MoCA is a 30-point screening tool designed to be highly sensitive in detecting mild cognitive impairment. It assesses multiple cognitive domains, including short-term memory recall, visuospatial and executive functions, attention, concentration, working memory, language, and spatiotemporal orientation. A total score of 26 or higher is generally considered to be within the normal range. Given its brevity and high sensitivity, the MoCA is a widely accepted instrument for assessing cognitive status in older people in both clinical and research settings (17).

### Interpersonal Reactivity Index (IRI)

Empathy was assessed with the Interpersonal Reactivity Index (IRI), a 28-item self-report questionnaire that yields four subscales (Perspective Taking, Empathic Concern, Personal Distress, and Fantasy). We used the total IRI score as a global index of dispositional empathy in line with prior work in older adults. The IRI has been adapted and validated across different cultures, including a Spanish version that has demonstrated psychometric properties similar to the original (18).

### Mind–body exercise program

Each weekly session lasted 60 min and was conducted indoors in small groups (8–12 participants) at CADI-UMAG. The instructor followed a standardized manual and checklist to ensure adherence. The fixed sequence comprised: 5 min focused on sitting and diaphragmatic breathing; 10 min of gentle joint mobility and dynamic warm-up; 20 min of multiplanar balance and gait sequences performed primarily standing, combining step patterns with coordinated upper limb movements and occasionally dual-task elements; 15 min of seated strengthening focused on lower limb and trunk muscles using body weight and resistance bands; and a 10-min cool-down that included slow breathing, body scan exercises, and brief mindfulness-oriented imagery. Intensity was maintained in a light to moderate range (Borg RPE 11–13), and progression was achieved by increasing range of motion, single-leg posture duration, and the complexity of coordination tasks.

### Statistical analysis

A Bayesian framework was employed to characterize changes in our variables of interest following the MBE intervention. Bayesian inference was preferred over traditional frequentist methods as it provides complete posterior distributions for all parameters, thereby allowing for a full quantification of uncertainty and a probabilistic interpretation via credible intervals (19).

Descriptive statistics were reported as mean and standard deviation (M ± SD) for continuous variables and as absolute and relative frequencies [*n* (%)] for categorical variables.

To measure pre-to-post intervention changes in the response variables, physical activity level (MET-min/week and sitting time), and cognitive level (MoCA score), we fitted Bayesian linear mixed-effects models. In these models, time (pre/post) was specified as the main fixed effect, adjusting for age, sex, and education level as covariates. Interactions between time and these covariates were also included to assess their influence on the intervention’s effect. Subject ID was included as a random effect to capture and isolate inter-individual variance from the main fixed effects.

Weakly informative priors, centered on a null effect, were used to exert a regularizing effect, reducing the influence of outliers and stochastic noise. All models were fitted using the No-U-Turn Sampler (NUTS) algorithm, a variant of Hamiltonian Monte Carlo (HMC). We ran 4 chains, with 10,000 warm-up iterations and 10,000 effective iterations per chain. Model convergence was confirmed by ensuring an *R*-hat statistic <1.01 and an effective sample size (ESS) > 2,000, and by visually inspecting trace plots to confirm convergence to a stationary distribution.

Results were reported as standardized beta coefficients ( β ) with 95% credible intervals (CI_95%_) as the measure of effect size, along with the probability of direction (pd) and the probability of significance (ps).

## Results

### Sample characteristics

The final enrolled sample in this study consisted of 44 older people (mean age of 69.7 ± 5.3 years), with 29 female and 15 male individuals. Sample characteristics can be observed in Table 2 and post-intervention descriptive statistics in Table 3.

**Table 2:** Descriptive statistics of the overall collected sample at their baseline measurements, aggregated for sex.

Characteristic	Overall *N* = 44	Female *N* = 29	Male *N* = 15	Difference	95% CI
Age (years)	69.70 ± 5.30	69.90 ± 5.22	69.33 ± 5.63	0.11	−0.52, 0.73
Body mass (kg)	78.03 ± 16.61	73.89 ± 14.51	86.59 ± 17.93	−0.80	−1.5, −0.14
Bipedal height (cm)	158.97 ± 8.49	156.07 ± 7.88	165.50 ± 5.93	−1.4	−2.1, −0.64
BMI (km/m^2^)	30.57 ± 5.67	30.16 ± 5.73	31.48 ± 5.66	−0.24	−0.92, 0.44
Education level				0.78	0.13, 1.4
No education	0 (0%)	0 (0%)	0 (0%)		
Primary education	11 (25%)	6 (21%)	5 (33%)		
Secondary education	22 (50%)	18 (62%)	4 (27%)		
Tertiary education	11 (25%)	5 (17%)	6 (40%)		
Sedentary time (minutes)	253.36 ± 165.68	250.90 ± 167.46	258.13 ± 167.89	−0.04	−0.67, 0.58
Physical activity (METs/week)	943.26 ± 917.79	770.21 ± 864.18	1,277.83 ± 954.76	−0.57	−1.2, 0.06
IRI total score	23.77 ± 3.05	23.21 ± 3.26	24.93 ± 2.27	−0.63	−1.3, 0.02
MoCA total score	22.66 ± 4.43	23.28 ± 4.09	21.47 ± 4.96	0.41	−0.22, 1.0
MoCA: viso-executive score	3.25 ± 1.53	3.31 ± 1.65	3.13 ± 1.30	0.12	−0.50, 0.75
MoCA: identification score	2.75 ± 0.58	2.72 ± 0.65	2.80 ± 0.41	−0.14	−0.77, 0.48
MoCA: attention score	4.57 ± 1.47	4.59 ± 1.48	4.53 ± 1.51	0.04	−0.59, 0.66
MoCA: language score	1.86 ± 0.90	1.83 ± 0.97	1.93 ± 0.80	−0.12	−0.75, 0.50
MoCA: abstraction score	1.48 ± 0.66	1.45 ± 0.63	1.53 ± 0.74	−0.13	−0.75, 0.50
MoCA: delayed recall score	3.09 ± 1.51	3.48 ± 1.35	2.33 ± 1.54	0.81	0.17, 1.5
MoCA: orientation score	5.66 ± 0.71	5.90 ± 0.31	5.20 ± 1.01	0.96	0.31, 1.6

Data is presented as mean ± standard deviation. Additionally, the standardized mean difference per sex and 95% confidence interval (CI) is presented for each variable.

**Table 3:** Post-intervention descriptive statistics for the main behavioral and cognitive outcomes, by sex.

Characteristic	Overall *N* = 44	Female *N* = 29	Male *N* = 15	Difference	95% CI
Age (years)	70.57 ± 5.83	70.56 ± 6.07	70.58 ± 5.73	0.00	−0.74, 0.73
Weight (kg)	78.57 ± 16.72	72.46 ± 13.30	87.73 ± 17.63	−1.0	−1.8, −0.24
Height (cm)	160.24 ± 8.23	155.59 ± 6.55	166.83 ± 5.41	−1.9	−2.8, −1.0
BMI (km/m^2^)	30.37 ± 5.62	29.56 ± 5.22	31.52 ± 6.20	−0.35	−1.1, 0.39
Education level				0.74	−0.01, 1.5
No education	1 (3.3%)	1 (5.6%)	0 (0%)		
Primary education	6 (20%)	3 (17%)	3 (25%)		
Secondary education	17 (57%)	12 (67%)	5 (42%)		
Tertiary education	6 (20%)	2 (11%)	4 (33%)		
Sedentary time (min)	173.40 ± 141.71	185.11 ± 164.62	155.83 ± 102.45	0.22	−0.51, 0.95
Physical activity (METs/week)	1,584.88 ± 1,385.49	1,128.33 ± 872.04	2,269.71 ± 1,741.49	−0.86	−1.6, −0.10
IRI total score	25.23 ± 4.15	25.56 ± 5.18	24.75 ± 1.86	0.21	−0.52, 0.95
MoCA total score	22.43 ± 4.22	22.44 ± 4.57	22.42 ± 3.82	0.01	−0.72, 0.74
MoCA: viso-executive score	3.77 ± 1.45	3.83 ± 1.76	3.67 ± 0.89	0.12	−0.61, 0.85
MoCA: identification score	2.77 ± 0.63	2.78 ± 0.73	2.75 ± 0.45	0.05	−0.68, 0.78
MoCA: attention score	4.07 ± 1.74	4.00 ± 1.61	4.17 ± 1.99	−0.10	−0.83, 0.64
MoCA: language score	1.87 ± 0.94	1.94 ± 0.87	1.75 ± 1.06	0.21	−0.52, 0.94
MoCA: abstraction score	1.57 ± 0.63	1.61 ± 0.50	1.50 ± 0.80	0.17	−0.56, 0.91
MoCA: delayed recall score	2.77 ± 1.63	2.56 ± 1.76	3.08 ± 1.44	−0.34	−1.1, 0.40
MoCA: orientation score	5.63 ± 0.67	5.72 ± 0.46	5.50 ± 0.90	0.32	−0.41, 1.1

Data is presented as mean ± standard deviation. Additionally, the standardized mean difference per sex and 95% confidence interval (CI) is presented for each variable.

### Effect on physical activity levels

When assessing the impact of the MBE-based intervention program (physical exercise and mindfulness), we observed overall improvements in physical activity–related indices. There was a reduction in time spent seated, used as a proxy of sedentary behavior [ β = − 0.71, CI_95%_ (−2.07, 0.52), pd = 86.1%, ps = 82.5%]. In parallel, we found a concurrent increase in weekly energy expenditure expressed as METs [ β = 0.52, CI_95%_ (−0.76, 1.81), pd = 78.9%, ps = 74.2%].

When examining covariates, age showed a negative association with seated time at baseline, indicating that younger participants tended to spend more time seated than older ones [ β = − 0.22, CI_95%_ (−0.58, 0.15), pd = 88.9%, ps = 75.2%]. Importantly, the interaction between age and time suggested that these younger, more sedentary individuals were also the ones who reduced their seated time the most over the course of the intervention [ β = 0.46, CI_95%_ (0.03, 0.89), pd = 98%, ps = 94.8%], Sex and education, in contrast, were not credibly related to seated time in our models.

**Figure 1:**
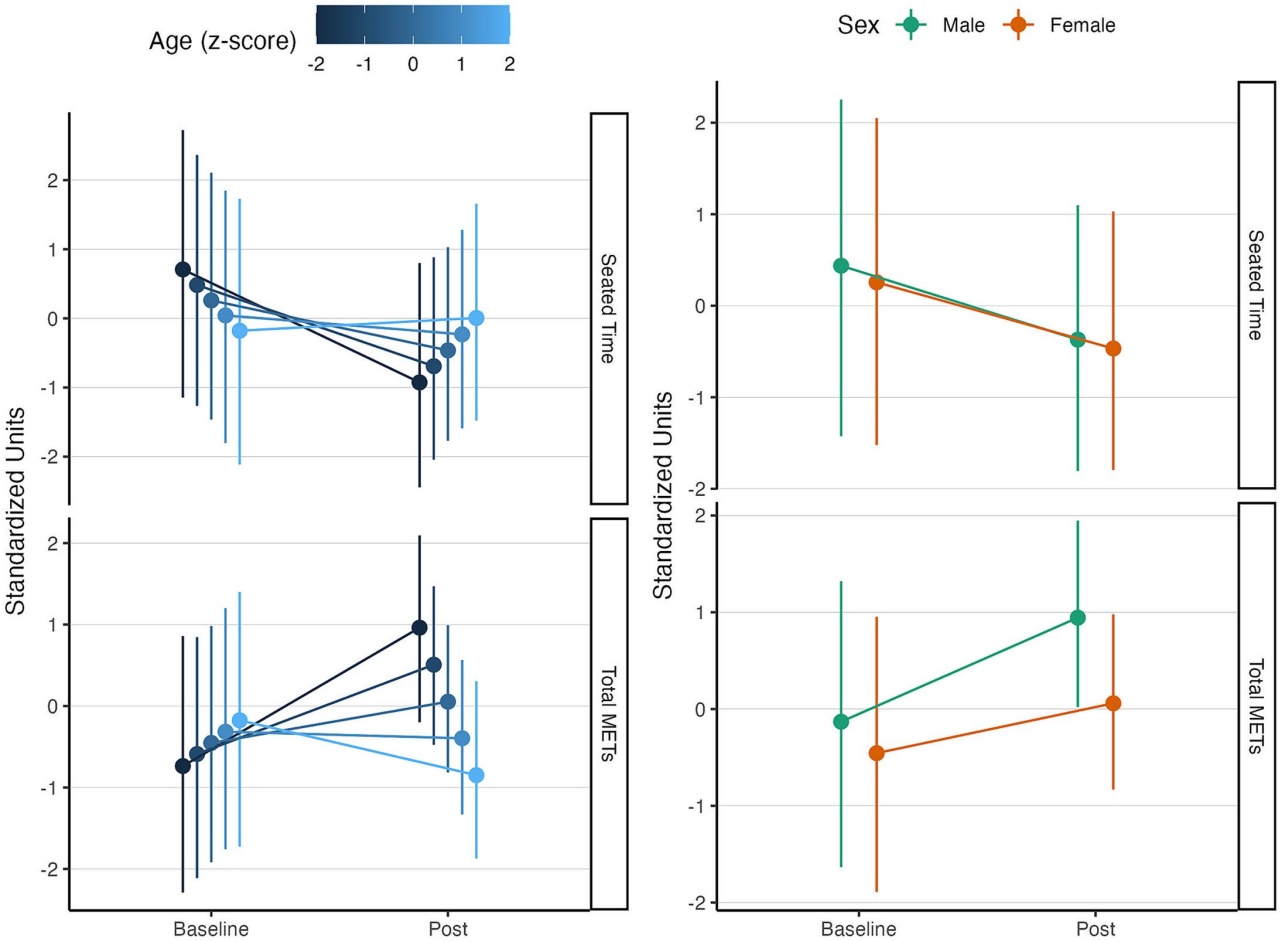
Effects of the intervention program on physical activity levels. Line graph illustrating the changes in the seated times, METs per week, and their temporal evolution in response to the physical exercise and mindfulness-based intervention program. All effects are displayed in standardized units. In the left panel, the age interaction effects are displayed, and in the right panel, the sex interaction effects are shown. The levels of age are displayed on a *z*-score. All effects are adjusted for confounders.

For physical activity (METs per week), age was positively associated with activity levels, such that older participants showed somewhat higher METs at baseline [ β = 0.14, CI_95%_ (−0.16, 0.44), pd = 83.8%, ps = 61.4%]. Additionally, older men tended to have higher physical activity levels than older women, reflected in higher METs per week [ β = 0.33, CI_95%_ (−0.34, 0.93), pd = 84.3%, ps = 75.7%]. Consistent with these patterns, exploratory interaction effects suggested that the main characteristics associated with increased energy expenditure after the intervention were being younger [*β* = 0.14, CI95% (−0.16, 0.44), pd = 83.8%, ps = 61.4%] and male [*β* = 0.33, CI95% (−0.34, 0.93), pd = 84.3%, ps = 75.7%], although the wide credible intervals indicate that these effects should be interpreted cautiously. Education did not exert moderating effects on any of the physical activity indices. These interaction effects can be observed in Figure 1.

### Effect on cognitive function levels

#### Total MoCA score

No significant changes were observed in the global MoCA score [ β = − 0.27, CI_95%_ (−1.63, 1.1), pd = 65.2%, ps = 60.1%]. As expected, age was associated with lower scores [ β = − 0.09, CI_95%_ (−0.42, 0.25), pd = 70.2%, ps = 47.3%], similar effect observed for male older people, scoring lower on average than female [ β = −0.34, CI_95%_ (−1.03, 0.38), pd = 83%, ps = 74.9%]. On the other hand, education was a potent positive driver of global MoCA scores [ β = 1.18, CI_95%_ (−2.05, 4.6), pd = 76.7%, ps = 74.7%]. The main interaction effects were linked to sex, whereas females older people experienced a more pronounced increase in global MoCA scores compared to male individuals [ β = 0.27, CI_95%_ (−0.71, 1.24), pd = 70.9%, ps = 63.4%], suggesting a sex-specific effect of MBA programs on cognitive function levels.

#### MoCA subdomains

In relation to MoCA subdomains, we observed different effects according to the assessed cognitive domain. Viso-executive domains showed a marked improvement after the intervention [ β = 0.45, CI_95%_ (−0.87, 1.78), pd = 74.5%, ps = 69.6%]. Negative influences of these improvements were observed due to age, with older people experiencing smaller increases in response to the intervention [ β = −0.26, CI_95%_ (−0.61, 0.09), pd = 92.1%, ps = 80.8%]. Positive influences were exerted by education levels, increasing the cognitive benefits of the intervention for more educated individuals [ β = 1.07, CI_95%_ (−2.12, 4.32), pd = 73.8%, ps = 71.8%].

Attention scores showed a small decrease relative to baseline [ β = −0.62, CI_95%_ (−1.93, 0.69), pd = 81.3%, ps = 77.1%], meaning slightly lower MoCA attention points after the intervention. Because higher MoCA scores indicate better performance and the credible interval was wide, we interpret this pattern as random intra-individual fluctuation or test fatigue rather than a systematic negative effect of the MBE program. In contrast, higher education was linked with an improvement, rather than a worsening, in the attention scores [ β = 1.03, CI_95%_ (−2.19, 4.14), pd = 73.2%, ps = 71%], positioning the individual’s education as a potent modulator of the cognitive effects of the MBE intervention. In addition to education, age was another driver of the response in attention scores. In contrast, younger individuals showed a less marked decrease in attention scores compared to older individuals [ β = 0.2, CI_95%_ (−0.33, 0.72), pd = 78%, ps = 65.2%].

In relation to language scores, no significant changes were observed [ β = − 0.04, CI_95%_ (−1.33, 1.24), pd = 53.1%, ps = 46.9%]. Moreover, male older people tend to have greater language scores at baseline [ β = 0.36, CI_95%_ (−0.38, 1.08), pd = 83.6%, ps = 75.4%]. However, they also displayed a worsening in language scores relative to baseline measurements, compared to their female counterparts [ β = −0.53, CI_95%_ (−1.48, 0.36), pd = 87.6%, ps = 82.9%].

**Figure 2:**
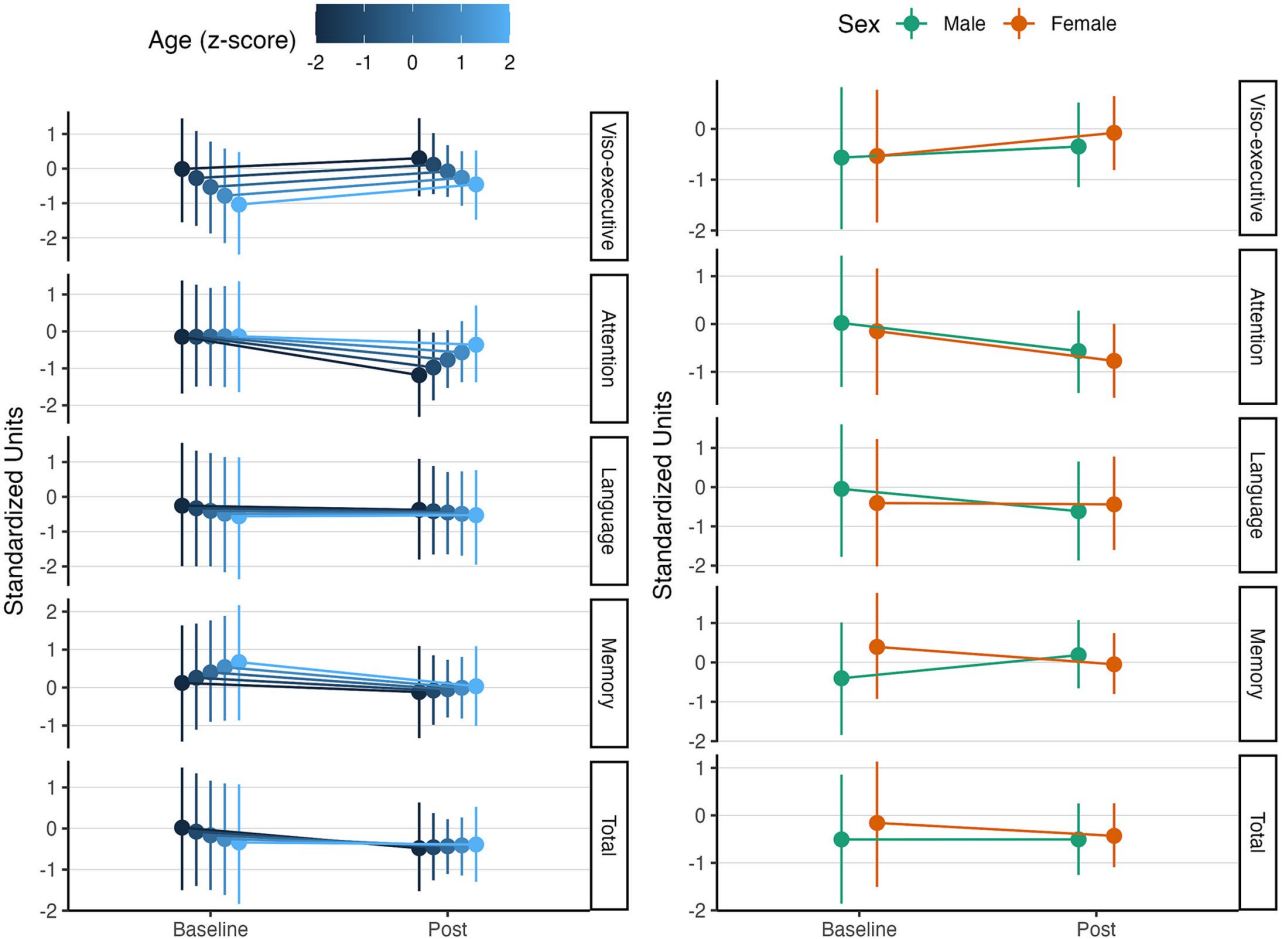
Effect of MBA intervention program on Montreal Cognitive Assessment (MoCA) scores. The line graph illustrates the changes in MoCA scores, linked to cognitive function and its evolution in response to a physical exercise and mindfulness-based intervention program. All effects are displayed in standardized units. The left panel illustrates the interaction effect between MBE intervention, MoCA scores, and age, whereas the right panel displays the interaction with sex. All effects are adjusted for confounders.

In regard to memory scores, linked to delayed recall abilities, we observed a decrease after MBE intervention [ β = −0.44, CI_95%_ (−1.78, 0.94), pd = 73.8%, ps = 68.9%]. However, this effect was reversed in males older people [ β = 1.03, CI_95%_ (−0.1, 2.13), pd = 96.5%, ps = 95.1%], despite of having lower baseline scores than females [ β = −0.8, CI_95%_ (−1.57, 0.04), pd = 97.4%, ps = 95.6%], suggesting a sex-specific effect in memory in response to MBE interventions. These effects can be seen in Figure 2.

### Effect on interpersonal reactivity

When assessing the MBE intervention’s effect on interpersonal reactivity, a positive effect was observed linked with an increase in IRI’s scores [ β = 0.59, CI_95%_ (−0.75, 1.94), pd = 81.2%, ps = 76.7%].

Similarly, a sex-specific effect was observed, whereas males tend to have greater baseline empathetic characteristics [ β = 0.61, CI_95%_ (−0.18, 1.38), pd = 93.9%, ps = 90.3%]. However, they tend to experience a decrease in this socio-affective feature in response to the MBE intervention compared to females [ β = −0.97, CI_95%_ (−1.92, −0.07), pd = 97.8%, ps = 96.7%].

**Figure 3:**
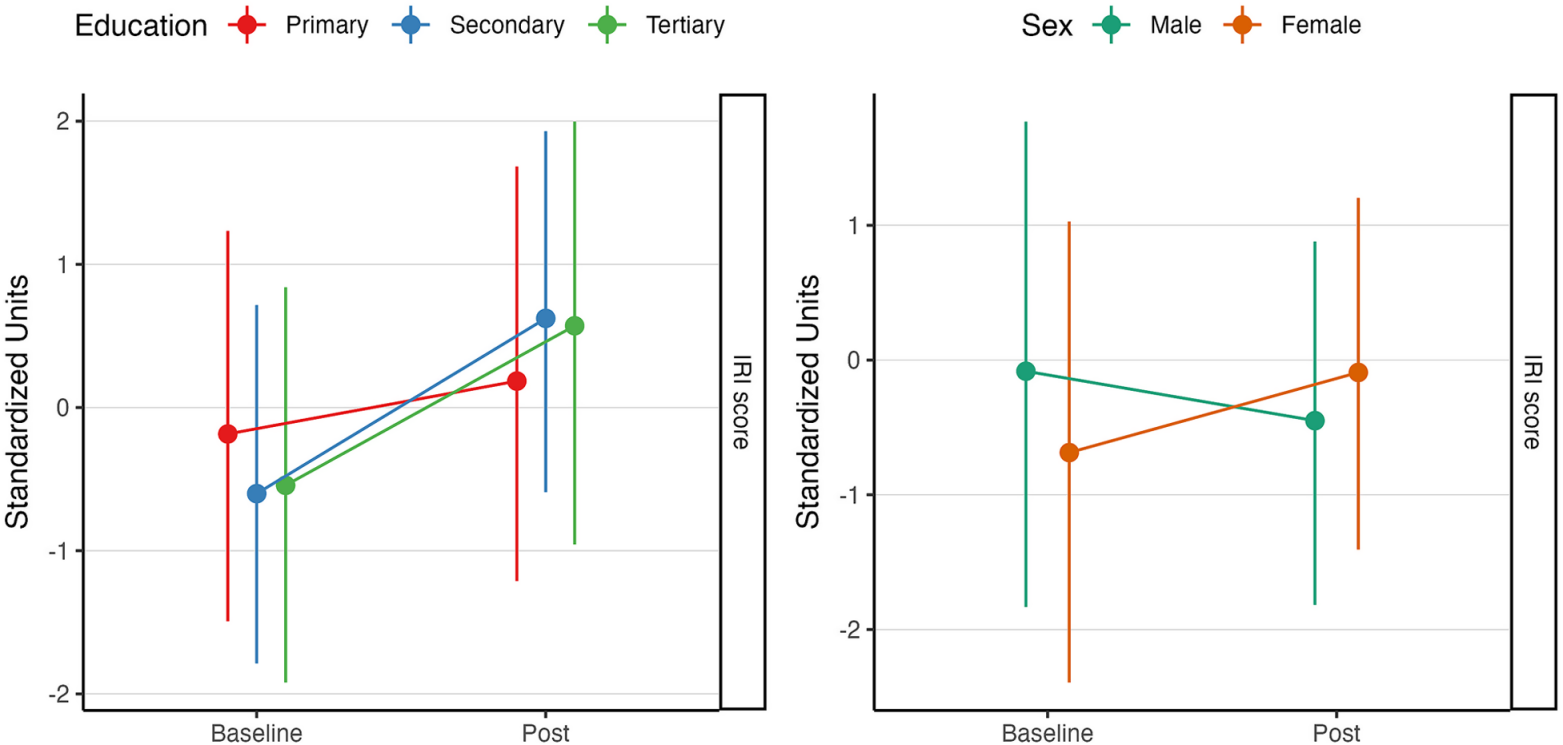
Effect of MBA intervention program on interpersonal reactivity index (IRI) scores. The line graph illustrates the changes in IRI scores, linked to empathetic attitude and its evolution in response to a physical exercise and mindfulness-based intervention program. All effects are displayed in standardized units. The left panel illustrates the interaction effect between MBE intervention, IRI scores, and education, whereas the right panel displays the interaction with sex. All effects are adjusted for confounders.

Further covariate analysis showed that those older people with higher education increased their post-intervention IRI scores in a linear fashion [ β = 1.14, CI_95%_ (−2.13, 4.48), pd = 75.1%, ps = 73.1%]. These effects can be seen in Figure 3.

## Discussion

This MBE program successfully reduced sedentary behavior and increased weekly physical activity level in older people. The program followed a fixed 60-min sequence that combined diaphragmatic breathing, joint mobility, multi-planar balance and gait tasks, chair-based strengthening, and a mindfulness-oriented cool-down, which jointly targeted postural control, attentional focus, and socio-affective engagement. These behavioral patterns coincided with a trend toward improved visuospatial–executive performance and increased empathy, while the global MoCA score was essentially unchanged. This specific cognitive enhancement is consistent with the known sensitivity of fronto-parietal and cerebellar circuits to interventions that integrate postural balance and breath control (4, 5, 20). We also observed a notable increase in empathy, measured by the IRI, which was moderated by sex and education. The pattern is compatible with prior reports that mind–body and multicomponent programs may preferentially affect executive and social-affective functions, but we did not measure neural activity or training intensity directly and therefore cannot make mechanistic claims about specific fronto-parietal or cerebellar pathways. Instead, we interpret the cognitive and socio-affective signals as hypothesis-generating indications that even a low-dose, climate-adapted program may be able to engage regulatory and social processes that are relevant for long-term adherence and community engagement (21).

The mixed cognitive profile observed here, numerical improvement in the visuospatial–executive subscore alongside small, non-significant decreases in attention and memory, must be interpreted with caution. All MoCA domain estimates were associated with substantial uncertainty, and the MoCA was not designed as a domain-specific cognitive outcome measure. It is therefore not possible to determine from our data whether the apparent domain differences reflect true specificity of training effects, regression to the mean, sampling variability, or state-related factors such as fatigue or motivation at testing. In line with existing literature on short, low-intensity interventions, we view these findings as preliminary and exploratory rather than confirmatory evidence of selective enhancement of visuospatial–executive function reliant on the fronto-parietal and cerebellar pathways (2, 3, 22). The focused improvement in visuospatial-executive function ( pd≈.75 ) in our sample strongly supports this view. In contrast, the slight, non-significant declines in attention and memory, which showed wide uncertainty, likely reflect intra-individual variability or assessment fatigue rather than a negative intervention effect (9). This pattern, where targeted executive and socio-affective gains precede changes in global cognition, is an expected outcome for short-term interventions (23, 24). Future trials using more granular cognitive batteries, higher training doses, and appropriate control groups will be required to clarify which domains are most sensitive to this type of program.

Our findings situate well within the broader literature. While meta-analyses confirm that longer, more intensive multicomponent exercise programs yield small-to-moderate cognitive benefits (25–27), others report inconsistent effects from specific practices like Tai Chi on executive tasks, underscoring the heterogeneity in the field (9). In this context, our results are both plausible and coherent. The intervention was potent enough to enhance sensorimotor integration and attentional control but too brief to shift the global cognitive score.

### Strengths and limitations

The study’s primary strengths are its standardized, regionally relevant indoor design, the novel inclusion of the IRI to assess social cognition, and a Bayesian analytical approach that offers a nuanced view of effect probabilities. However, several limitations warrant emphasis. First, the training dose was intentionally minimal (one 60-min session per week), which is below current recommendations for older adults and limits the extent to which our findings can be generalized as a stand-alone public-health intervention. Second, there was no control group, intervention duration was relatively short, and we relied exclusively on self-report measures (IPAQ) to characterize physical activity and sedentary time, which contributes to wide uncertainty in dose–response inferences. Third, we did not quantify exercise intensity or cumulative workload (via heart rate, accelerometry, or session ratings of perceived exertion) and did not include objective biomarkers such as heart rate variability or sleep measures, nor did we stratify by season, a critical factor in the high-latitude Magallanes region. Taken together, these limitations preclude causal conclusions and any strong claims about physiological mechanisms, but they do not negate the convergent, low-intensity signals we observed across behavior, selective cognition, and social cognition.

We also did not collect objective data on sleep, heart-rate variability, or blood pressure, nor did we use wearable devices to continuously track daily activity. Incorporating accelerometry, actigraphy, and health-monitoring watches in future studies would strengthen causal inference and replication in this high-latitude context.

### Practical applications

Our findings suggest that once-weekly (60 min) indoor MBE may function as a feasible, low-barrier entry point or adjunct within primary geriatrics, particularly in settings where climatic conditions and resource constraints limit access to higher-dose exercise programs. In practice, such a program could be offered to older adults with low-to-moderate IPAQ levels and high sitting time, using a brief, safe screening (MoCA and basic functional tests) to ensure suitability. The standardized session structure (breathing, mobility, balance, light resistance, chair-based options) and use of checklists can support fidelity and ≥80% adherence, while simple monitoring of MET-min/week, sedentary minutes/day, sensitive cognitive subdomains, and a brief empathy measure every 4–8 weeks can provide clinically useful feedback. Importantly, we envision this type of MBE as one component in a broader, multi-modal strategy to reduce sedentarism and support healthy aging, rather than as a complete replacement for guideline-recommended volumes of physical activity.

### Future research directions

Building on these preliminary findings, we propose a clear roadmap for future research. (i) Conduct extended trials of at least 16–24 weeks, with three or more sessions to 60 min per week, is necessary to confirm long-term effects (26, 27). These trials should stratify participants by season and education level to control for key moderators. (ii) Incorporate objective and more granular measures, since future studies must integrate objective data from accelerometry (physical activity level), heart rate variability (autonomic function), and actigraphy (sleep behavior). The IRI should also be analyzed at the subscale level (e.g., Perspective-Taking, Empathic Concern) to create detailed response profiles and identify mediators of change. (iii) Establish transferable dose–response protocols, whereas the ultimate goal is to define dose–response thresholds and progression criteria that can be implemented effectively in community and primary care settings, accounting for local climate and seasonality.

## Conclusion

This study provides preliminary evidence that a standardized, once-weekly MBE program, tailored to high-latitude climatic constraints, may be associated with a favorable but modest pattern of change in older adults: reduced self-reported sedentary behavior, potential sharpening of executive control, and enhanced social-affective functioning. Given the low training dose, small sample, and self-reported outcomes, these findings should not yet be interpreted as definitive or as evidence of a fully scalable public-health solution. Rather, they highlight the feasibility and acceptability of a climate-adapted mind–body format and offer dose and design parameters that can inform more intensive, rigorously controlled trials aimed at supporting prevention and functional independence in extreme environments.

## Data Availability

The raw data supporting the conclusions of this article will be made available by the authors, without undue reservation.
